# On the Fury Fits of the Elephant

**Published:** 1831-01-01

**Authors:** 


### LVII.

### ON THE FURY FITS OF THE ELEPHANT.

[Phrenological Journal.]

IT is well known that the half-reasoning elephant is, in general, a docile and inoffensive animal. Yet sometimes, and often without insult or injury, this powerful creature will scatter destruction in every direction; witness the paroxysm of fury into which MADEMOISELLE D'JECK lately flung herself, to the dismay of all her keepers, and the death of a casual intruder. This orgasm, which does not depend on any apparent exciting cause, and is unconnected with the instinct of the sex, occurs when the animal is regularly supplied with food —when it has received, and is receiving the kindest treatment. In short, it appears to be a momentary fit of insanity. In India, when this rabies is manifested, it is allowed to subside, which it does of itself. We had a late example at Exeter 'Change, where the destruction of a noble animal was necessary to prevent the most dreadful consequences. More recently that entertaining performer at the Adelphi and other places, is said, in the Phrenological Journal, to have "killed one of her attendants." The same Journal observes that "as it has been said that the person had used her ill in some way, and had only been punished retributively for the offence, and there are no accounts of the animal's 'running a muck,' in a fit of indiscriminating ferocity, we are not at liberty to consider the Morpeth incident as of the same kind with that which produced the tragedy at Exeter 'Change." From circumstances which it is unnecessary to mention, we can inform the writer in the Phrenological Journal, that it was *not* one of the keepers that was killed by the ingenious quadruped comedian, but a person not usually in attendance, who happened to go into Miss D'Jeck's chamber, and make some noise. The lady seemed actuated by a sudden and quite unaccustomed burst of fury. She struck the intruder first with her trunk, and kindling into still greater rage, followed up her revenge, or rather madness, by dashing one of her tusks into his chest, and goring him against the wall of her chamber. The paroxysm of fury was soon over, and she manifested no disposition to injure her keepers.

We are informed by Mr. Corse Scott, who long had the charge of the Company's elephants in India, that these animals are subject to paroxysms of the above description. "He states that he has seen an elephant so excited suddenly gore other elephants with its tusks, and kill them outright by transfixing them to the ground." In a recent number of the Library of Entertaining Knowledge, we find the following passage bearing on this subject.

"We have already mentioned an instance of the ferocity of the elephant under a peculiar state of excitement, as observed by Mr. Corse. This state is indicated in both sexes, *and is probably in some degree relieved* by the secretion of a brownish juice from a considerable gland at the temple, through an opening in the skin,' (I would say excited by the secretion, and relieved by the exudation.) 'The aperture is situated between the ear and the eye, on *each* side of the head, and the gland is immediately under the skin on each side also. The glands are as much as six inches in diameter, but the aperture is scarcely perceptible. This peculiarity is noticed by Strabo; and the Indian mythology has seized upon the circumstance for one of its fanciful devices. The Hindoo poets often allude to the fragrant juice which oozes, at certain seasons, from small ducts in the temples of the male elephant' (Mr. Corse says of the female, too,) 'and is useful in relieving him from the redundant moisture with which he is then oppressed; and they even describe the bees as allured by the scent, and mistaking it for that of the sweetest flowers."

We are told by the writer in the Phrenological Journal, that "there is always a great and continued exudation (from the aperture above-mentioned) during a fit of fury, and for some time after it; while it appears that the fit is moderated and ultimately allayed by the discharge." As the gland from which this secretion issues is, he believes, situated near, if not immediately over the organ of destructiveness, he queries whether a state of inflammation in the gland may not act in exciting the portion of contiguous brain, and account for the temporary fit of homicidal insanity. We agree with him that the subject is worthy the attention of our Asiatic phrenologists, as one which may tend to throw some light upon the "instinct of destructiveness as a specific primitive impulse, having its organ in the brain."

The paroxysm of the Adelphi performer, though transient, appears to have been allied to those above described. We have made inquiries, but cannot learn that there was any exudation of the kind alluded to by Mr. Scott. The elephant has gone on a voyage to our transatlantic brethren, to whose amusement she will no doubt contribute. They need be under no alarm respecting the young lady's organ of destructiveness; for we can assure them that, excepting on the above occasion of an untimely intrusion on her privacy, she has not manifested any propensity to combativeness, much less destructiveness, during all her travels and performances from the banks of the Dwina to the pillars of Hercules—and even to ULTIMA THULÉ.

---

